# Designing Organoid Models to Monitor Cancer Progression, Plasticity and Resistance: The Right Set Up for the Right Question

**DOI:** 10.3390/cancers14153559

**Published:** 2022-07-22

**Authors:** Flora Doffe, Fabien Bonini, Emile Lakis, Stéphane Terry, Salem Chouaib, Pierre Savagner

**Affiliations:** 1 INSERM UMR 1186, Integrative Tumor Immunology and Immunotherapy, Gustave Roussy, Faculty of Medicine, University Paris-Saclay, 94805 Villejuif, France; flora.doffe@gustaveroussy.fr (F.D.); stephane.terry@gustaveroussy.fr (S.T.); salem.chouaib@gustaveroussy.fr (S.C.); 2 Department of Pathology and Immunology, Faculty of Medicine, University Geneva, 1205 Geneva, Switzerland; fabien.bonini@unige.ch; 3 LGC Standards, 67120 Molsheim, France; emile.lakis@lgcgroup.com; 4 Thumbay Research Institute for Precision Medicine, Gulf Medical University, Ajman 4184, United Arab Emirates

**Keywords:** organoid, 3D model, microfluidics, preclinical assay, plasticity, extracellular matrix, breast cancer, resistance, personalized medicine

## Abstract

**Simple Summary:**

In this article, we describe a 3D model that supports the maintenance of cell polarity in cancer and normal cells by growing them on a collagen-coated microsupport. Instead of the spheroids model, the cells are directly positioned to adopt a basal/luminal organization, favoring differentiation and migration in the surrounding matrix. This model can be enriched with other components of the microenvironment such as fibroblasts and immune cells. For the proof-of-concept experiments, we treated mouse and human cell lines, then primary tumor cells from PDX, co-cultured with fibroblasts or immune cells. We monitored cell viability, proliferation and cytotoxicity using several light emission-based methods to obtain significant and reliable results, validating the method.

**Abstract:**

The recent trend in 3D cell modeling has fostered the emergence of a wide range of models, addressing very distinct goals ranging from the fundamental exploration of cell–cell interactions to preclinical assays for personalized medicine. It is clear that no single model will recapitulate the complexity and dynamics of in vivo situations. The key is to define the critical points, achieve a specific goal and design a model where they can be validated. In this report, we focused on cancer progression. We describe our model which is designed to emulate breast carcinoma progression during the invasive phase. We chose to provide topological clues to the target cells by growing them on microsupports, favoring a polarized epithelial organization before they are embedded in a 3D matrix. We then watched for cell organization and differentiation for these models, adding stroma cells then immune cells to follow and quantify cell responses to drug treatment, including quantifying cell death and viability, as well as morphogenic and invasive properties. We used model cell lines including Comma Dβ, MCF7 and MCF10A mammary epithelial cells as well as primary breast cancer cells from patient-derived xenografts (PDX). We found that fibroblasts impacted cell response to Docetaxel and Palbociclib. We also found that NK92 immune cells could target breast cancer cells within the 3D configuration, providing quantitative monitoring of cell cytotoxicity. We also tested several sources for the extracellular matrix and selected a hyaluronan-based matrix as a promising alternative to mouse tumor basement membrane extracts for primary human cancer cells. Overall, we validated a new 3D model designed for breast cancer for preclinical use in personalized medicine.

## 1. Introduction

Relying on clinically relevant functional screening for new pharmaceutical compounds is not yet an option in oncotherapy. Classic 2D cell culture screening, appropriate for high-throughput studies, provides very disappointing results when used to predict clinical evolution [1]. In vivo testing is not much better in terms of clinical relevance: About 80% of the drugs selected from animal studies lack efficiency when tested clinically [2]. Clearly, there is a strong need for more relevant models to monitor essential cellular and immune responses during cancer progression, before and after treatment. Recently, the 3D cell culture revolution has raised hopes for better assays, offering a more realistic organization for tumor microenvironment and a more relevant functional monitoring covering cell organization [3] and polarization [4]. These models should analyze apoptosis induction in cancer cells [5] as well as invasion, estimated by different methods [6]. Culture medium should not interfere with the progression and be potentially enriched in growth factors mimicking stroma impact by slow-release systems [7]. These models must eventually be compatible with high-throughput screens [8,9,10]. Recent advances in immunotherapy have raised new hopes in the development of more efficient drug treatments. They have also emphasized the need to design 3D models compatible with the introduction and monitoring of immune cells [11,12]. To provide the 3D topology, an initial extra-cellular matrix mesh must be present, which will become enriched and reorganized by the cells as they grow from inside. Classic biomaterial such as collagen I and basement membrane extracts (BME) can be used for this purpose, but a large choice of more reliable synthetic biomaterials based on polyethylene glycol (PEG), polyethylene glycol-poly vinyl alcohol (PEG-PVA) and other derivatives are now easily accessible, including functionalized biomaterials harboring cell adhesion sites for integrins [13,14,15,16,17].

The goal of this reconstituted microenvironment is to provide cells with a physical and biochemical environment that will support mesenchymal cell differentiation and tumor cell plasticity, organization and interactions [18,19]. Typically, numerous pathways are involved in these processes. Defining which ones are relevant in the context of a specific tumor type will be key to selecting an appropriate combination of inhibitors, for a personalized therapy strategy.

In this report, we introduced a new 3D model, designed to analyze breast tumor cell response to treatment and immune cell exposure. We used current clinical drugs and NK cells to elucidate the difference between 2D and 3D conditions. We monitored cell viability, invasiveness and cytotoxicity in mammary carcinoma cell lines and patient-derived xenografts (PDX)—tumor cells in several matrices.

## 2. Materials and Methods

### 2.1. Cell Culture

The Comma Dβ cell line [20] was derived from the parental Comma D cells and graciously provided by Dr. Medina (Baylor college of Medicine, Houston, TX, USA) who generated them. They were grown in DMEM/F12 (Gibco, Asnières-sur-Seine, France) supplemented with 2% FCS (Gibco), 10 μg/mL bovine insulin (Sigma, Saint-Quentin-Fallavier, France) and 5 ng/mL murine EGF (Sigma, Saint-Quentin-Fallavier, France). Cells were routinely subcultured at split ratios of 1:5. MCF-10A cells were purchased from the American Type Culture Collection (ATCC, Manassas, VA, USA) and grown in DMEM/F12 (Gibco, Asnières-sur-Seine, France) containing 5% horse serum, 20 ng/mL epidermal growth factor, 0.5 μg/mL hydrocortisone, 0.1 μg/mL cholera toxin, 10 μg/mL insulin (Sigma, Saint-Quentin-Fallavier, France) and 1% penicillin-streptomycin solution. Cell identity was confirmed by STR profiling. MCF7 were purchased from the ATCC. They were grown in DMEM (Gibco, Asnières-sur-Seine, France supplemented with 0.8 nM β-Estradiol, 10% FBS (Gibco, Asnières-sur-Seine, France) and 1% Penicilline/Streptavidine (Gibco, Asnières-sur-Seine, France) Immortalized GFP-labeled human fibroblasts were generated by A. Turtoi (IRCM, Montpellier, France) who kindly made them available to us. They were cultured in DMEM (Gibco, Asnières-sur-Seine, France) supplemented, 10% FBS (Gibco, Asnières-sur-Seine, France and 1% Penicilline/Streptavidine (Gibco, Asnières-sur-Seine, France). NK-92 were purchased from ATCC. They were grown in RPMI (Gibco) + Penicilline/streptavidin 1% (Gibco, Asnières-sur-Seine, France) FBS 10% (Gibco, Asnières-sur-Seine, France and 200 U/mL IL-2 (Sanofi, Paris, France, IAI, Lod, Israel).

### 2.2. PDX Processing

PDX_BRE-IGR-0134 primary cells originate from a docetaxel-treated breast luminal B tumor grade 3, stage pT4, stage pN3, ER PR positive and HER2 negative. It has been transplanted and left to grow until it reaches 1500 mm^3^ on 3 passages by the preclinical evaluation platform at Gustave Roussy. The tumor was collected and processed in our laboratory. Tumor dissociation was adapted from [21]. Upon arrival, the tumor tissues were weighed and washed with PBS. The tumor was cut in order to isolate two random pieces of 1–3 mm^3^ that were fixed in formalin for histopathological analysis and immunohistochemistry 24 h at 4 °C. The remaining tissues was minced in a Petri dish with a scalpel and directly digested in an enzyme mix (Tumor Dissociation Kit human, Miltenyi, Bergisch Gladbach, Germany). The samples were incubated in a 37 °C incubator under continuous rotation for 40 min. Evaluation of the viability was carried out with trypan blue. If the sample was necrotic, dead cells was removed by magnetic sorting (dead cell removal kit, Miltenyi) following the manufacturer’s instructions. Live cells were kept in a supplemented medium (Table S1), but experiments were performed with neither inhibitors nor growth factors present in the supplemented medium. The simplified medium is composed of DMEM (Gibco, 41965-039), 10% fetal bovine serum (Gibco, A3840402), 1% penicillin streptavidin (Gibco, 15140-122) and 0.8 nM β-Estradiol (Sigma, E-2758).

### 2.3. Microsupport Culture

After trypsinization, five million cells were resuspended in 5 mL culture medium, then incubated with 3 mL Cytodex Microsupports (Cytodex 3, 17-0485-01, SIGMA, resuspended at 0.01 g/mL), before adding 42 mL culture medium in a bioreactor on a magnetic rotary stirrer in a 50 mL final volume. Cells were incubated until 50–80% confluency was reached (about 48 h).

### 2.4. Drug Treatment

Alpelisib (HY-15244, Medchem Express) was used at 1 µM; Palbociclib (PZ0383, Sigma) was used at 1 µM in DMSO; and Docetaxel (Docetaxel Accord, Gustave Roussy pharmacy, Villejuif, France) was used at 20 nM in Ethanol. Dose was based on prior 2D assays (Figure S1) and Gustave Roussy clinicians’ expertise.

### 2.5. MTT Assay

Thiazol Blue Tetrazolium Bromide (M5655-1G, Sigma-Aldrich, St. Louis, MO, USA) was diluted in DMEM without phenol red (21041-025 Gibco) (Cf = 1 mg/mL), the solution filtered at 0.45 µm (051230, Dutsher). The cell medium was removed and cells were incubated for 1 to 3 h with 100 µL of MTT solution (1 mg/mL) at 37 °C. MTT solution was then removed and 100 µL 2-Propanol (20,842.298 1L VWR) was added per well. After 10 min of shaking at 190 rpm, necessary to solubilize the Formazan formed (blue-violet), plates were scanned with a spectrophotometer at 570 nm.

### 2.6. Cytotoxicity Assay

Cells were co-cultured with the NK-92 cells embedded in the collagen I/BME mix. Cell ratio was 1:1 for MCF7 cells and 5:1 NK92 for MCF10A cells. The cells were co-cultured for 24 h to one week. The cytotoxicity assay was achieved with the CellTox Green Dye (Promega, Madison, WI, USA) following the manufacturer’s instruction. The reagent was added to the culture medium. Incubation lasted for 15 min, shielded from light, and measurement was completed with a microplate reader fluorescence (485 Excitation/520 Emission, Spectramax i3×, Molecular Devices, San Jose, CA, USA).

### 2.7. Organoid Model

The collagen (354249, Corning, Corning, NY, USA)—BME (basement membrane extract, 3445_005_01, Cultrex, Trevigen, Gaithersburg, MD, USA) matrix was prepared by diluting collagen to a concentration of 2 mg/mL in PBS 1× (Gibco). pH was neutralized with a 10× solution of HEPES (22 mg/L, Gibco) and sodium bicarbonate (48 mg/mL, Sigma) and supplemented with a 10× solution of DMEM (Life Technologies, Carlsbad, CA, USA). The BME is diluted in the collagen at 1.53 mg/mL. A first layer of matrices is left to polymerize at the bottom of the well for 20 min, then overlayed with matrices as well as Cytodex beads loaded with cells at the same volume of the first layer in a Lab-Tek Chamber Slide system (8 wells, Sigma). After polymerization (15–30 min at 37 °C), the medium was added on the top of the gel. Alternatively, we used a hyaluronate-based matrix (Hystem-HP, Sigma) for MCF10A cells. The medium was changed every other day and cells were allowed to grow for 48 h to two weeks as indicated.

### 2.8. Histology Block Preparation

BME/collagen gels containing cells on microsupports were fixed in a 4% paraformaldehyde solution for 45 min at room temperature. Then, the block was included in a 3% agarose gel followed by a post-fixation in a 4% paraformaldehyde solution before paraffin embedding, processed by the PETRA histology technological platform (Gustave Roussy). Slides were sectioned with a microtome (5 μm sections), then deparaffinized and processed for antigen retrieval by incubation in citrate buffer (pH 6) at 100 °C for 1 h. For thicker sections (100 μm), vibratome was used, and in this case, the gels were not embedded in paraffin, but kept in PBS-azide (0.1%).

### 2.9. Immunofluorescence Staining

Slides were incubated for 1 h at room temperature with blocking buffer (10% heat inactivated goat serum, 0.3% Triton X-100 in PBS). Incubation with primary antibodies (1:100) was performed for 1 h at room temperature (thin sections) or overnight at 4 °C (thick sections). Then, the slides were washed three times with PBS + Tween 20 (0.1%). Next, incubation with secondary antibodies was carried out for 1 h at room temperature (1:750) and DAPI (1:1000). Non-specific staining was blocked by adding 10% of the goat serum to primary antibodies.

Antibodies were obtained from several sources: Keratin 5 (PRB160P, BioLegend, San Diego, CA, USA), Keratin 8 (MMS-162P, BioLegend), α6 Integrin (313602, BioLegend), mucin1 (Rabbit Abcam), E-cadherin (13-1700, Zymed, San Diego, CA, USA), Desmogleins (SAD-3121, Labgen) and PCNA1 (2586S CST, caspase 3 (9662, CST).

### 2.10. Microscopy

For imaging, pictures and movies, a IX83 microscope (Olympus, Tokyo, Japan) was used with proprietary analysis software (CellSense Dimension, Olympus). Some pictures were taken with a multiphoton microscope (SP8, Leica) from the PFIC platform (Gustave Roussy).

## 3. Results

### 3.1. A Composite Model to Optimize Functional Monitoring

**Figure 1:**
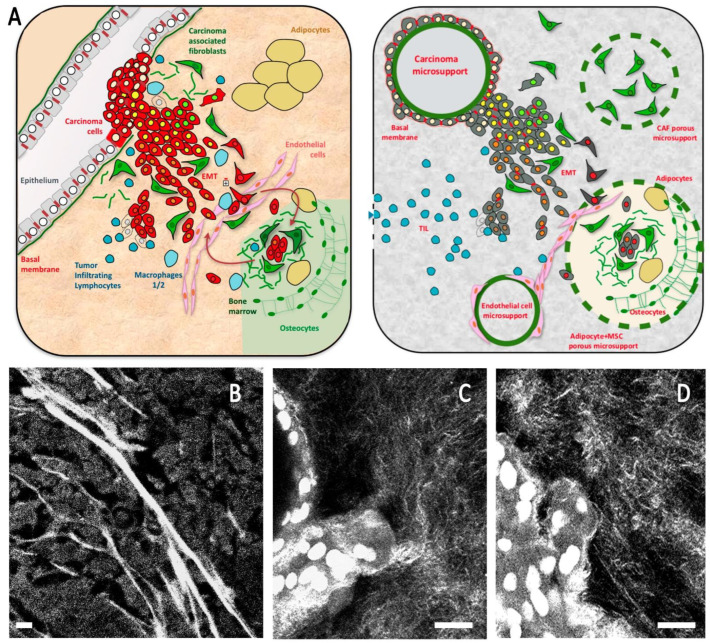
(**A**) Schematic breast cancer microenvironment including the main cell types present in the microenvironment and the composite model design to emulate this organization. (**B**–**D**) Second harmonic imaging showing organized collagen mesh, including thick fibers in the breast tumor (**B**) and a thinner mesh in composite organoid model including a high proportion of collagen 1 (**C**,**D**). Scale bar: 20 µm (**B**), 10 µm (**C**,**D**).

Our model is based on a simpler 3D method that we published in 2018 to report the impact of several siRNA on the emergence of 3D tubule-like processes by mammary epithelial cells [22]. The rationale for this method is to generate a polarized epithelial layer, emulating mammary tubules in terms of providing a mechanical support and a migration orientation, similar to in vivo situations observed during development and tumor progression on histological slides (Figure 1). 

Our goal is to analyze breast tumor cell response to treatment and immune cell introduction. We chose a configuration favoring tumor cell growth and morphogenesis, to be monitored and quantified over one to two week periods. Cells are directly confronted to the surrounding extra-cellular matrix (ECM), sometimes enriched with stroma cells.

**Figure 2:**
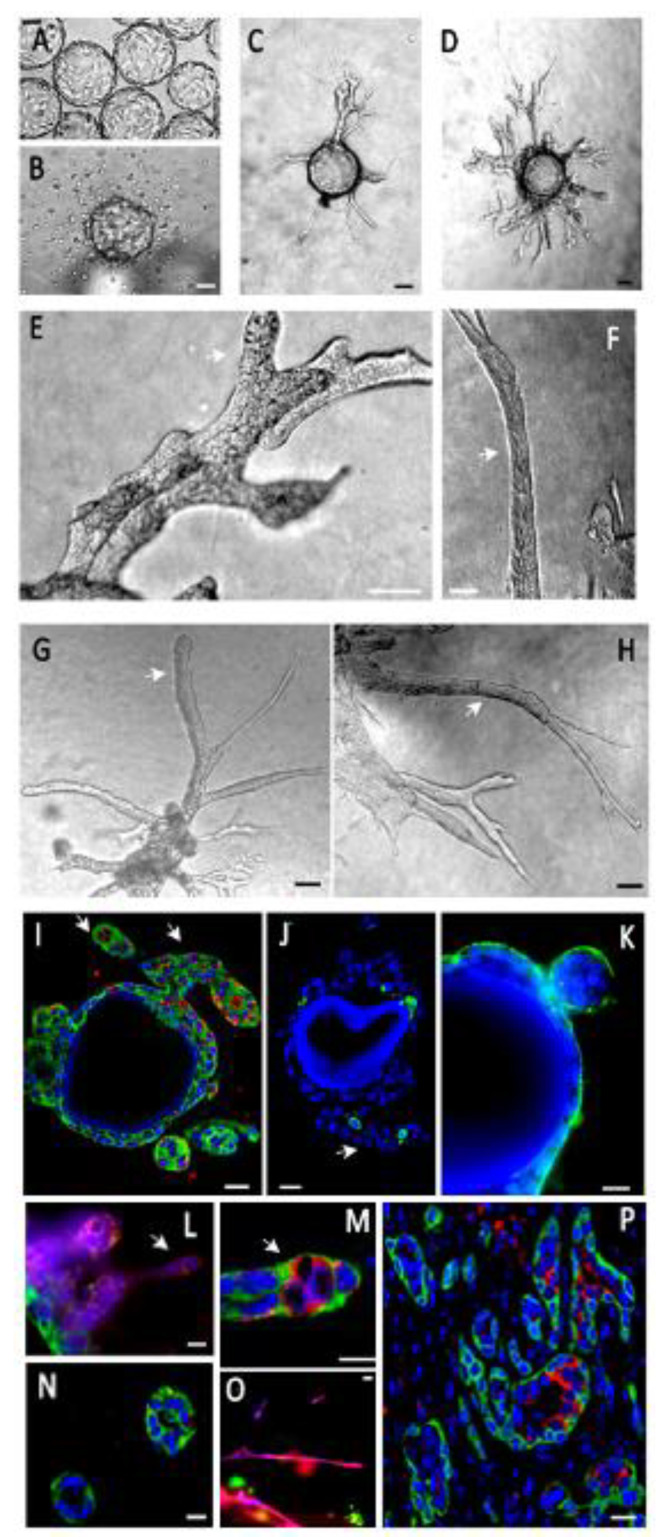
Mouse mammary epithelial cells (Comma Dβ) express tubule-like processes in our organoid model. (**A**)—Cells were grown in suspension on Cytodex beads. (**B**)—In a collagen I gel, cells left the beads and scattered individually. (**C**)—In a collagen I/BME gel, they initiated tubule-like processes after 48 h, migrating upright from the bead surface. (**D**)—This organization was stable for at least 2 weeks, involving the thickening and branching of these processes. (**E**–**H**)—The tubule-like processes (white arrows) were very cohesive and organized. (**I**)—Epithelial cells expressed CK5 (green) and PCNA1 (red), demonstrating a basal phenotype and an active proliferation (PCNA1+) more intense in invading processes (white arrow). (**J**)—Apoptosis, as estimated by caspase 3 expression (green), was only found in a small percentage of cells (about 5%), typically in the center of tubule-like processes. (**K**)—Epithelial cells covering the microsupports or budding expressed basement membrane receptor integrin α6 (green) at the outer edge, indicating a polarized organization. (**L**–**O**)—Invading processes were composed of basal (CK5, green) and luminal (CK8, red) cells. Basal cells (red) were cohesive and facing gel (**M**,**N**). Luminal cells (CK8, red) migrated in looser, thinner structures or as individual cells (**L**,**O**). They were also found inside the thicker tubule-like processes, facing an emerging lumen, reconstituting their physiological organization in mammary tubules (**M**). (**P**)—This cell organization was very similar to the configuration observed in vivo upon the subcutaneous injection of Comma Dβ cells (CK5 green, CK8 red). Scale bar: 10 µm.

We used several cell models, in addition to primary tumor cells from PDX. We first tested mouse Comma Dβ immortalized mammary epithelial cells. These cells possess several features of stem/progenitor cells: they can regenerate a full mammary gland structure including all epithelial subtypes in vivo when injected in situ [23]. We grew them on non-porous bead microsupports, until they reached confluency. The first step was growing the Comma Dβ cells on microsupports in suspension on a rotary shaker, until the surface of the beads was covered up to 80% by the spreading cells (Figure 2A). Cell-covered beads were then embedded in a matrix composed of mixed collagen I (2 mg/mL) and BME (1.5 mg/mL) and left to grow for a week or more as cohesive groups or individualized cells and establish a tumor-like microenvironment. Choice of the collagen/BME was a pragmatic compromise used by other laboratories [24]. Fibrillar collagen favored spontaneous isolated cell dissociation from the beads, with fewer or no tubule-like process emergence (Figure 2B). Introducing BME resulted in reduced cell motility, enhanced epithelial differentiation and eventually tubule formation. BME alone promotes cell differentiation but diminishes morphogenesis and tubule-like emergence (data not shown). Escaping cell groups migrate perpendicularly to this cell layer (Figure 2C) to invade the composite ECM, under gradually increasing pressure resulting from cell proliferation and underlying rigid substrate (beads). By providing a physical starting point, this configuration also helped us design a more comprehensive method to quantitate invasiveness and epithelial layer stability. Typically, the migratory process was initiated by one cell and began after 48–72 h, when the cells actively proliferated and became overconfluent at the surface of the microsupport, generating increasing pressure on the ECM (Figure 2C–H).

Comma Dβ cells expressed basal and luminal cell markers (CK5 for basal-myoepithelial cells, and CK8 for luminal cells) and were able to invade the ECM from the microsupports by establishing very coherent, organized and partially differentiated tubule-like structures (Figure 2D–H, Video S1). Few apoptotic cells could be found by caspase 3 immunostaining. We observed that 5.63% cells expressed caspase 3, based on the screening of 195 cells from two independent sections. After one week of growth, proliferation was stronger in the invasive processes with 51% PCNAI + cells (in a sampling of 67 cells from invasive processes) versus 10% in the peri-microsupport area (among 50 cells from the basal layer) based on three independent sections (white arrowheads, Figure 2E–I). These invasive processes were composed of basal (CK5, green) and luminal (CK8, red) cells. Basal cells (red) were cohesive and facing gel (Figure 2M,N). Luminal cells (CK8, red) migrated in looser, thinner structures or as individual cells (Figure 2L,O). After tubule-like structure thickening and reorganization, luminal cells expressing luminal marker cytokeratin 8 (CK8) were identified inside the growing tubule-like structures, mimicking the physiological organization with a basal/myoepithelial cell layer expressing cytokeratin 5 (CK5) (Figure 2M) and basement membrane receptors (α6 integrin) surrounding a layer of luminal CK8+ facing the lumen. This organization was very similar to what we observed in vivo, after injecting Comma Dβ cells in mice subcutaneously (Figure 2P). 

We also used this model for the immortalized human mammary epithelial cell line (MCF10A) and breast cancer cell line MCF7. MCF10A express a mostly basal phenotype, including a limited CK8+ sub-population within a largely predominant CK5+ population when MCF7 cells express a luminal phenotype mostly expressing CK8.

**Figure 3:**
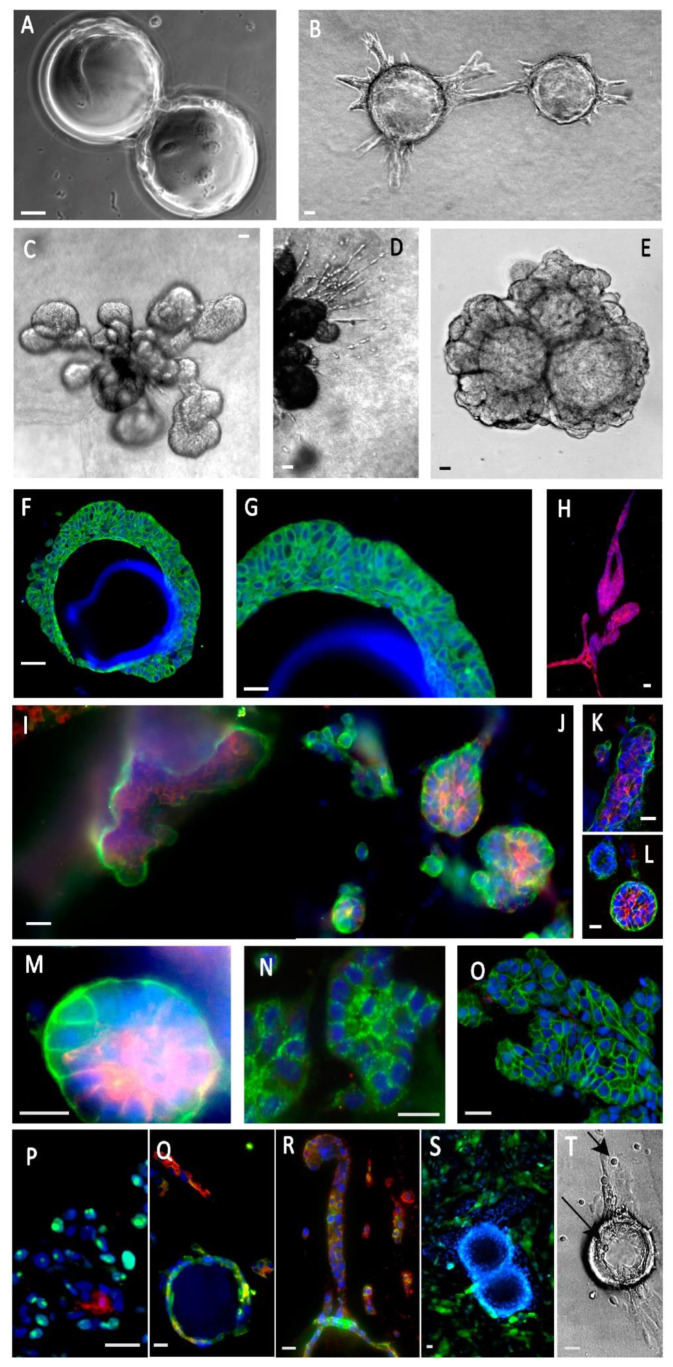
Human mammary epithelial cells (MCF10A) express partly differentiated structures in our composite organoid model. (**A**)—Cells were grown in suspension on Cytodex beads. (**B**)—When embedded in a collagen/BME hydrogel, they initiated organized tubule-like structures. (**C**)—These structures gave rise to globular buds. (**D**)—Some cells migrated as individual cells or cords, among the bigger tubule-like processes. (**E**)—Other gel compositions, such as a hyaluronate/gelatin hydrogel, induced less active migration, but allowed organized morphogenesis and formation of organized buds stable after a one-month culture. (**F**,**G**)—Cells formed overlapping layers at the surface of the beads (CK5, green). (**H**)—A subpopulation of luminal cells (CK8, green) could be seen, migrating as individualized cells. (**I**–**M**)—Cell polarization was demonstrated by the basal localization of α6 integrin (green) and the luminal localization of mucin 1 (red). It should be noted that the same cells coexpress α6 integrin and mucin 1. (**N**)—Desmosome expression is attested by the cell edge punctuated localization of desmogleins (green). (**O**)—Cohesive cell buds express E-cadherin at cell edges (green). (**P**)—Bud cells expressed PCNA1 (green), suggesting active proliferation and a low level of apoptosis, always inside cell buds (caspase 3, red). (**Q**,**R**)—MCF10A cells adhering to the microsupports expressed CK5 (green), but lost it to CK8 (red) in cell chords migrating away from the beads. (**S**)—Introducing GFP-labeled fibroblasts induced narrow cell–cell interactions. (**T**)—Introducing immune NK cells (NK92) resulted in invasive migration and epithelial cell binding by NK cells (arrows). Most immunofluorescence panels included Hoechst dye to visualize nuclei. Scale bar: 10 µm.

**Figure 4:**
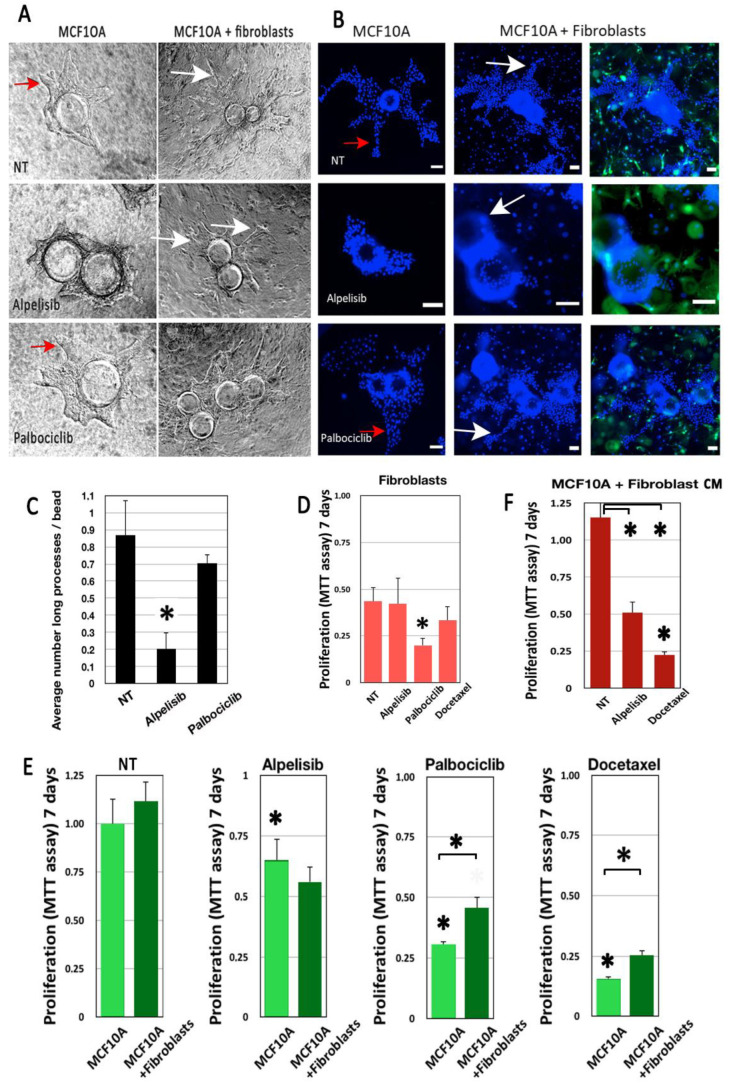
Drug impact is modulated by fibroblasts in our morphogenic model. MCF10A cells were grown in a collagen I/BME hydrogel with or without a GFP-labeled mammary fibroblast. MCF10A grew invasive processes that invaded the 3D matrix, visible in phase contrast (red arrows, **A**) or after Hoechst dye staining (red arrows, **B**). Alpelisib treatment inhibited the growth of these processes significantly (**A**–**C**). However, fibroblast addition enhanced the size and complexity of these invasive processes in all cases (white arrows). Fibroblasts alone were not sensitive to the doses of treatment used for MCF10A, except for Palbociclib (**D**). Adding fibroblasts had no effect on untreated MCF10A viability, enhanced the impact of Alpelisib, but significantly protected the MCF10A from Palbociclib and Docetaxel treatment (**E**). Fibroblast-conditioned medium had the same impact as co-cultured fibroblasts on Alpelisib and Docetaxel treatment (**F**). Scale bar: 100 µm. * for *p* < 0.05 in a Student T test comparing treated with untreated cells. * with brackets is for *p* < 0.05 in a Student T test comparing columns as indicated.

Both cell lines grow on the microsupports (Figure 3A). Once embedded in the collagen/BME mixed matrix, these cells cover the microsupport with several) layers of cells. Eventually, MCF10A cells evade as isolated cells or tubule-like cohesive buds (Figure 3B–D,F–H, Figure 4A,B, Video S2) that maintain a basal polarity in contact with the ECM (Figure 3I–M). Interestingly, isolated cells and cohesive buds show different differentiation patterns. Isolated cells express luminal markers (CK8+) when the cohesive structures express predominantly basal markers (CK5+) (Figure 3Q,R). We could not detect a clear basal-luminal organization inside the invading structures, as seen with the Comma Dβ mouse cell line (Figure 2). Overall, apoptotic activity was low in this model, based on caspase 3 expression (2.55% on a 409 cells sample) compared to 18% estimated in 2D conditions earlier [25]. Proliferation was more intense in invasive processes seen in Figure 2 and Figure 3 as estimated by PCNA1 immunostaining (76% PCNAI + cells among 164 cells), compared to the peri-microsupport area (9.54% calculated from 241 cells from 3 independent sections).

In addition to the collagen I/BME mix, we also tested a hyaluronan/gelatin matrix (Hystem-HP), providing a better defined and reliable matrix. MCF10A cells grew and proliferated actively in Hystem-HP, progressively invading the ECM as an organized process, without significant cell–cell dissociation (Figure 3E). With improved transparency, cell organization and progression were visible by phase contrast microscopy (Figure 3E). The slow progression process was highly stable, at least for a one-month culture (Figure 3E), favoring drug testing as described in the next section.

**Figure 5:**
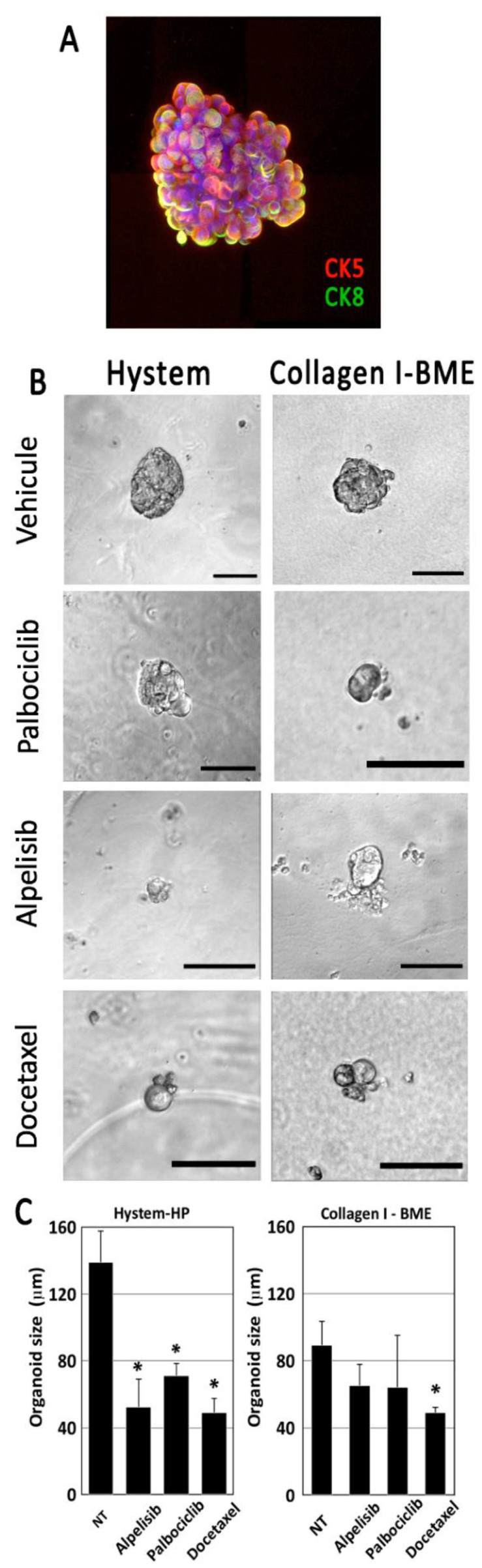
Primary tumor cells from patients were dissociated, reaggregated and cultured in Hystem-HP or collagen I/BME mixed hydrogel. Dissociated tumor cells were able to reaggregate in suspension, forming heterogeneous spheroids expressing both CK5 and CK8 (**A**). When cultured in Hystem-HP, aggregates grew slowly, but maintained their viability and phenotype over several weeks. They were sensitive to drug treatment, showing signs of cell death and reduced aggregate size as calculated from 35 spheroids (**B**,**C**). The Hystem-HP substrate was more favorable to tumor cell growth in this tumor. Scale bar: 100 µm. * for *p* < 0.05 in a Student T test comparing treated with untreated cells.

Finally, we isolated cells from a luminal B breast cancer sample from a patient treated with Docetaxel in order to grow them in our model. The tumor was dissociated to obtain small groups of tumor cells. Cell suspension was treated to eliminate most mesenchymal cells, and left to self-organize in suspension for 48 h, resulting in epithelial cell spheroids that we found by immunofluorescence to express cytokeratins 5 and 8 in a poorly organized mode configuration (Figure 5A). Cells clumps adopted a spheroid morphology after 48 h. They adhered poorly to the microsupport surface and we transplanted them as spheroids directly into hydrogels. We tested both collagen I/BME mix and Hystem-HP. Both matrices supported cell growth, but little invasion was seen after one to two weeks of culture. We tested drugs in both conditions to monitor viability and morphological response.

### 3.2. Drug Treatment

Several drugs used routinely to treat breast cancer were tested in 2D and 3D conditions for one week. We used Docetaxel, a chemotherapy drug used to treat metastatic breast cancer, but also more recently for adjuvant and neo-adjuvant treatment (before surgery). The tumor used to establish the PDX in these experiments used in these experiments was treated with Docetaxel at 20 nM. We also applied drugs used for personalized medicine such as Alpelisib, a PI3Kα inhibitor, and Palbociclib, a CDK4/6 inhibitor, targeting proliferation both at 1 μM. To complete our stroma component, we introduced immortalized fibroblasts into our model to evaluate their impact on drug responsiveness in terms of cell viability and invasiveness (Figure 4).

By phase contrast microscopy and fluorescent microscopy, we found that the inclusion of fibroblasts was clearly associated with an increase in the number and complexity of pseudo-tubules (Figure 4A,B). Alpelisib treatment significantly reduced the growth of these processes (Figure 4C) in MCF10A 3D cultures. However, the addition of fibroblasts restored the increased morphogenic progression (Figure 4A,B).

All drugs significantly affected MCF10A cell viability, as estimated by the MTT assay (Figure 4F, asterisks on first columns). Adding fibroblasts had a limited but significant impact on MCF10A survival under Palbociclib and Docetaxel treatment (Figure 4E, asterisks with bars). To analyze fibroblast impact, we also cultured MCF10A cells with conditioned media from untreated fibroblasts. Conditioned media provided some protection against Docetaxel treatment, similar to live fibroblasts, suggesting the putative involvement of soluble factors (Figure 4F).

Compared to the MCF10A, fibroblasts cultured alone in the same conditions were less affected by drug treatments, except for the Palbociclib treatment (Figure 4D).

We finally screened the same drugs on the breast luminal B PDX explants treated for one week. PDX spheroids were transplanted into two distinct matrices: our collagen I/BME mix or the Hystem hydrogel described previously. All cells were found to express cytokeratins, including cytokeratin 8, a specific marker for luminal B breast cancer (Figure 5A). Cells were treated for 7 days before monitoring explant growth, based on image analysis (Figure 5B). We obtained a more sensitive drug response in Hystem-HP compared to the BME mix (Figure 5C). In both cases, Docetaxel, used as a positive control, induced significant cytotoxicity visible on the phase contrast pictures (Figure 5B).

### 3.3. Immune Cells and Immunotoxicity

We selected a human NK cell line (NK92) for proof-of-concept experiments. NK92 cells are widely used for research purposes and are being evaluated in various indications as an attractive source of adoptive cancer immunotherapy [26].

**Figure 6:**
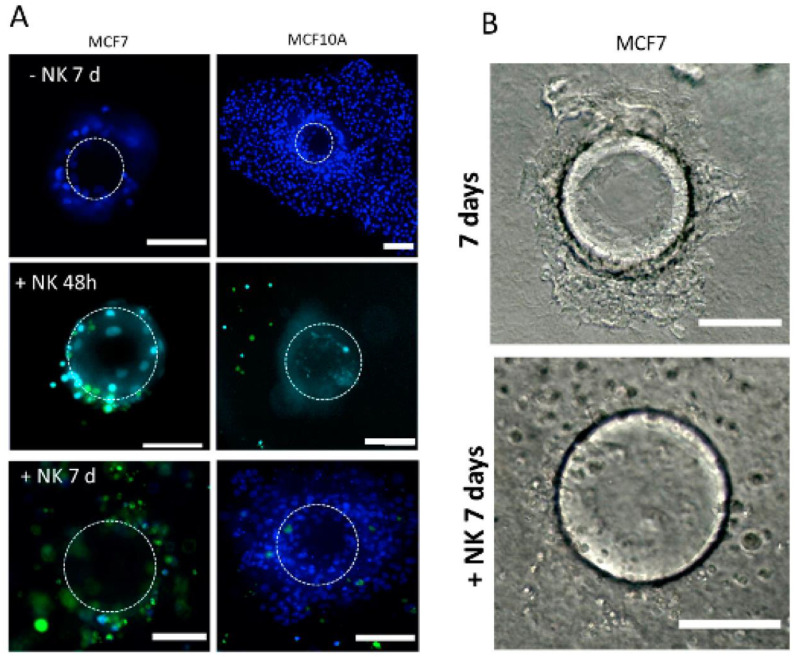

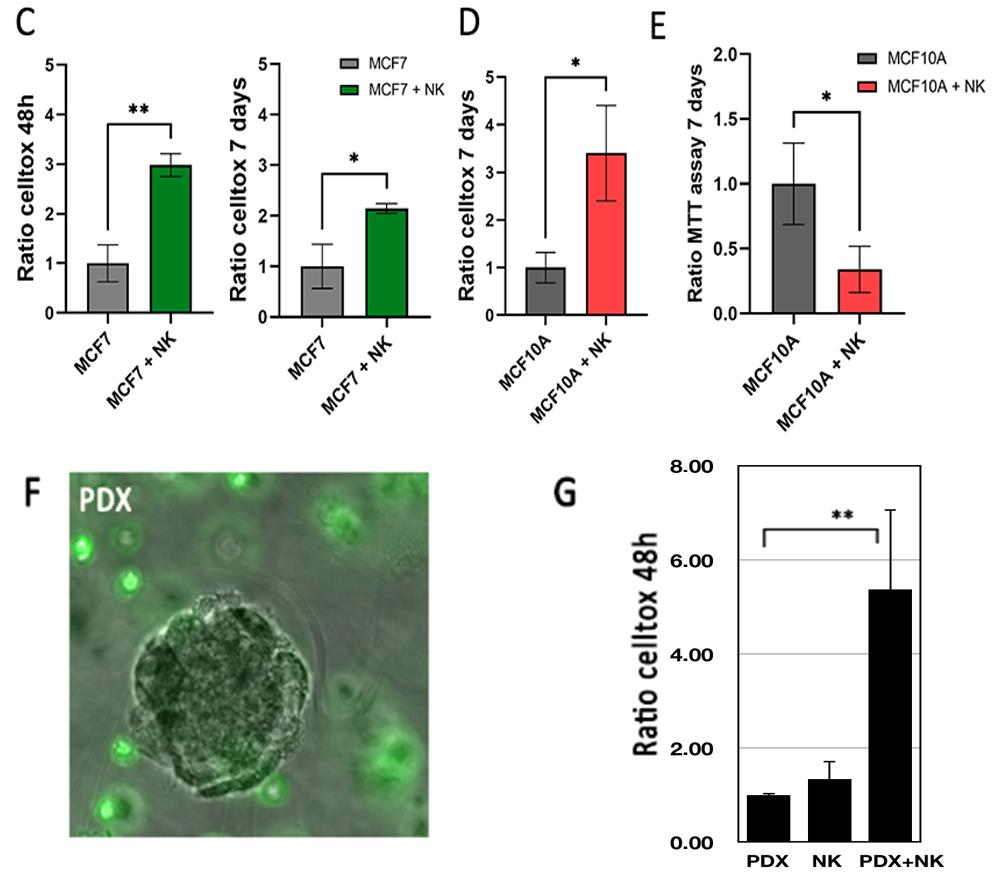
Immune cells NK-92 can detect and kill tumor cells in our models. (**A**–**C**) MCF7 and MCF10A cells were cultured on a microsupport in a collagen I/BME mix for 48 h and 7 days with and without NK-92 cells, stained with green CFSE. Dotted circle indicate microsupport location. All nuclei were stained with Hoechst dye (**B**) MCF7 cultured on a microsupport after 7 days with and without NK-92 in bright field. (**C**) Cytotoxicity was evaluated using Celltox on MCF7 after 48 h and 7 days. (**D**) Cytotoxicity was evaluated using Celltox on MCF10A after 7 days. In addition, MTT assay was performed on MCF10A after 7 days. Scale bar: 100 µm. (**E**) MCF1O1 were also tested for proliferation using MTT assay, with or without NK cells. (**F**) PDX explants were immersed in a collagen I/BME mix with stained NK-92 cells. (**G**) Cytotoxicity was evaluated by Celltox after 48 h for PDX cells and NK92 cells, isolated or combined. * for *p* < 0.05 in a Student T test comparing columns as indicated. ** for *p* < 0.001.

We found NK92 cells to actively migrate in collagen/BME, but not significantly in the hyaluronate matrix. They are able to interact and actively kill a significant proportion of MCF7 cells, as found after 48 h using a cytotoxicity assay (Figure 6). After 7 days, the NK cells had almost completely cleaned up the microsupport beads, remaining in vicinity (Figure 6C,D). Conversely, MCF10A cells did not show evidence of cytotoxicity at 48 h (Figure 6A). However, they went through massive NK-induced cell death after 7 days (Figure 6D) as evidenced by cell toxicity (Figure 6E) and MTT assays. The addition of NK cells was associated with a net decrease in cell viability in MCF10A after 7 days (Figure 6G). Finally, we tested PDX explants, immersed in a collagen I/BME mix. We found that the introduction of NK92 cells resulted in a strong and significant cytotoxicity, whereas each cell type alone only displayed a limited amount of cytotoxicity (Figure 6F,G).

## 4. Discussion and Conclusions

In this manuscript, we described a novel composite 3D model designed to monitor cell viability, cell death and invasion under drug treatment and immune response in a 3D environment including stroma elements. We tested immortalized and transformed cell lines to finally adjust our method for primary PDX-derived cells. The overall goal is to provide a preclinical tool for new drug screening, suitable for personalized treatment.

### 4.1. Proliferation and Migration

One of the main advantages of our microsupport model is to expose proliferative areas and invasive processes, documenting the emergence of tumor heterogeneity. Comma Dβ cells growing around the microsupports showed a proliferation index of 10%, (estimated by PCNAI expression). This is the proliferation level in classic subconfluent 2D conditions [25]. However, invasive processes were found to express significantly more PCNA I cells (50%). In MCF10A cells, the ratio was even higher. This feature from our model is supported by our design, providing a rigid starting position (microsupport bead) for migratory processes. The activation of integrins, extracellular matrix and motility in the control of cell proliferation is well known during development [27]. Here, we describe a similar phenomenon, already evoked in organoids in different models [3]. It seems clinically relevant to determine in vitro if tumor explants conserve morphogenic potential, and how this can be linked to proliferation and progression, potentially providing prognostic value and relevant therapeutic targets.

### 4.2. The Matrix Dilemna

We found that the stroma composition controlled morphogenesis and invasiveness, but was also critical in deciding monitoring methods, often based on a certain level of transparency. We selected two substrates, based on commercial components: the first one was a mix of collagen I and BME (Cultrex), favoring morphogenesis, cell motility. But this mix has a limited stability over time due to the contractility of the collagen fiber mesh, specially in presence of fibroblasts. The second substrate (Hystem) offered a simpler and better-defined composition, based on recombinant and crosslinked hyaluronans, enriched with gelatin, proving close interactions with a cell integrin apparatus. We found this second matrix to be more stable, paving the way for long-term experiments beyond one month. It was also a more transparent matrix, allowing us to follow morphology and organization more closely. Tested cells mostly remained cohesive, adopting a “rosette group phenotype” around the microsupport (Figure 3E), with cell groups coordinating a very slow invasive process, leading to invasive buds in MCF10A after several weeks to one month. 

BME have been extensively used due to their strong impact on cell survival and differentiation in 3D models. However, beyond the cost and the current shortage, several limits hamper the use of BME for 3D organoid models [28,29]. Reproducibility issues have been a problem from the start: different commercial lots have significantly different biological impacts. Additionally, BME hydrogels do not provide a stiffness comparable with most solid tumors, even at the highest protein concentration available, and do not reflect a physiological molecular organization, since it does not include a 3D-organized basement membrane. Indeed, clinical assays based on tumoroids embedded in BME do not provide an acceptable clinical prediction so far, for most drug treatments as observed by several authors [30,31,32,33,34]. For example, a study based on 12 patient-derived organoids was consistent with clinical evolution when treated with irinotecan, a DNA topoisomerase inhibitor. However, it did not show any predictive value for classic chemotherapy drugs such as 5-FU, a thymidylate synthase inhibitor, or oxaliplatin, a DNA complexing drug [35]. A compromise was found by combining collagen I and BME, bringing down the proportion of BME. This type of matrix does not reflect a physiological ECM but is much more efficient in promoting morphogenesis by providing both migratory and differentiation clues to the cells [36]. This method, used in our model, provides a method to evaluate morphogenetic potential, linked to invasiveness. We also used Hystem-HP, a recombinant hyaluronan-based matrix, including denatured collagen (gelatin). This gel offered a higher stiffness and transparency, supporting image analysis methods. This is particularly important, considering most live monitoring methods for cell responses, viability, death or metabolic status use light emission as an output. The hyaluronate-based matrix also provided a strong stability and proliferation potential with regards to our longer-term cultures.

### 4.3. Monitoring Drug Treatment

We found that drug impact was modulated by coculturing fibroblasts. Fibroblasts supported MCF10A viability partially but significantly under Palbociclib or Docetaxel after 7 days of treatment. Interestingly, adding fibroblast-conditioned media also attenuated the Docetaxel effect, with a light but significant pattern. These results suggest that at least some of the fibroblast impact is mediated by secreted factors. The role of fibroblasts in the cancer microenvironment is complex and involves several types of cancer-associated fibroblasts [37,38]. In further work, it may be necessary to recover them from patient biopsies and sort them specifically before co-culturing and treatment. Some models include slow-release designs, such as gelatin microspheres [6] to provide cells or growth factor gradient within the microenvironment. The addition of growth factors that mimic mesenchymal cells emphasizes the dilemma of culture medium in monitoring drug response in activated tumor cells.

Incidentally, it is important to note that most 3D tumoroid protocols, based on the protocols initiated in H. Clevers’s lab [35,39] for colon or breast organoids, include inhibitors of the P38MAPK, RhoK and TGFβ pathways for efficient cell differentiation and growth. Beyond the potential interest in maintaining cell differentiation status, this creates a serious problem if testing drugs that target these pathways in the context of personalized medicine [4]. We used these additives for the first steps of dissociation and recovery phases, but avoided inhibitors for the drug testing experiments.

### 4.4. A New Paradigm: The Introduction of Immune Cells

Recently, the development of new immunotherapy strategies has created a strong need for in vitro assays designed to directly test new drug impacts on immune response. This exploding area implies that 3D configuration is compatible with immune cell introduction, involving microfluidic elements [11]. The source and type of immune cells is the first challenge, dictated by the goal and tumor type. Several immune cell lines are now available. These models can target immediate response and immunotoxicity in short-term evaluations [38] or provide more complex environments, involving patient-derived immune cells. During the tumor cell dissociation process, it is possible for example to isolate lymphocyte (TIL) populations to test their potential afterward in the assay, with or without treatment. In our case, we found that a NK cell line expressing all classic NK markers was able to migrate, locate and kill target cells such as MCF7, with a significant impact after 48 h. Surprisingly, another putative target cell, MCF10A, was only significantly targeted after one week of co-culturing with NK92, suggesting that some maturation mechanisms were involved.

## 5. Conclusions

In conclusion, we tested a new model to document drug impact on tumor progression, using primary tumor cells from PDX. The choice of matrix was dictated by the selected functional monitoring. The presence of fibrillar collagen was found to be necessary to follow and monitor tumor cell invasive progression, providing an invasive pattern similar to the in vivo situation. The use of a crosslinked hyaluronate hydrogel provided a very stable matrix, supporting 3D cell growth and progression with a better level of transparency appropriate for direct live image analysis. To the best of our knowledge, this is the first report of primary tumor cells tested for drugs in a commercial hyaluronate-based matrix with a defined composition. Future research will examine primary tumors from patients for potential resistance in this model and the predictive value of these preclinical assays for personalized medicine.

## Data Availability

Data are contained within the article or Supplementary Material.

## Supplementary Materials

The following supporting information can be downloaded at: https://www.mdpi.com/article/10.3390/cancers14153559/s1, Supplemental Figure S1: MCF10A cells were cultured in 2D classic plates and treated for one week with Alpelisib and Palbociclib. Viability was evaluated by MTT assay at several concentrations. Values are reported to the drug diluent (DMSO). Supplemental Table S1: Breast cancer organoid culture medium. Video S1: Comma Dβ mammary epithelial cells were grown in a mixed matrix, on microsupports for two weeks. Cells reached a stable configuration state, with strong cell dynamics along the microsupport and tubule-like processes. Some cells appear to migrate in the matrix, joining each other for transient structures that could consolidate into the same tubule-like processes. Video S2: MCF10A were grown on microsupports for two weeks. By combining photos at different focus levels, this video shows the emergence of a distinct but minor cell subpopulation composed of isolated cells and loosely associated chords arising among the massive buds.
