# Supplementary figures and images for: Designing Organoid Models to Monitor Cancer Progression, Plasticity and Resistance: The Right Set Up for the Right Question
Source: Cancers (Basel). 2022 Jul 22;14(15):3559. doi: 10.3390/cancers14153559 (PMC9330027; doi:10.3390/cancers14153559)

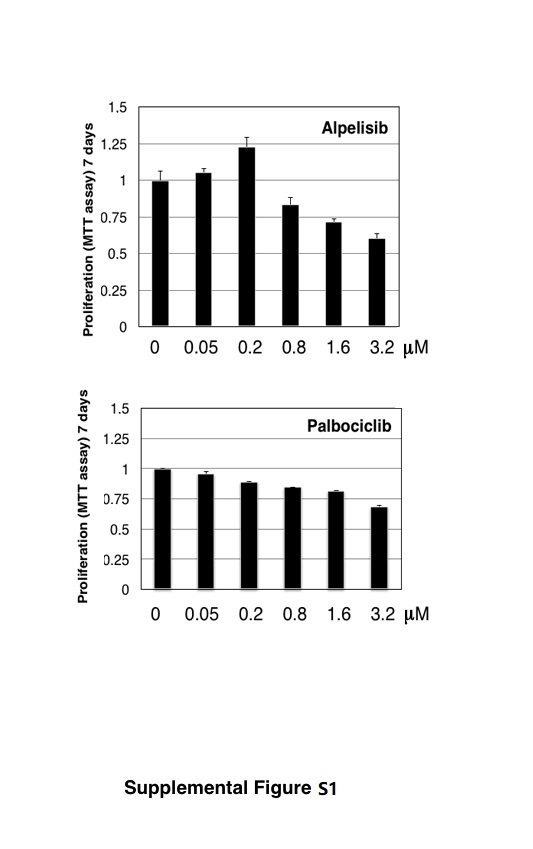

Supplement: Supplementary file 1 [file cancers-14-03559-s001.zip › Sup Fig S1.jpg]
